# Refractory Hypercalcemia Secondary to Metastatic Parathyroid Carcinoma Treated With Immunotherapy

**DOI:** 10.1210/jcemcr/luae127

**Published:** 2024-07-15

**Authors:** Brenda Ta, Michael James Bennett

**Affiliations:** Department of Endocrinology and Diabetes, Prince of Wales Hospital, Randwick 2031, Australia; Department of Endocrinology and Diabetes, St Vincent's Hospital, Darlinghurst 2010, Australia; School of Medicine, UNSW, Kensington 2033, Australia; Department of Endocrinology and Diabetes, Prince of Wales Hospital, Randwick 2031, Australia; School of Medicine, UNSW, Kensington 2033, Australia; Department of Endocrinology, The Sutherland Hospital, Caringbah, NSW 2229, Australia; The Garvan Institute of Medical Research, Darlinghurst, NSW 2010, Australia

**Keywords:** metastatic parathyroid carcinoma, immunotherapy, nivolumab, refractory hypercalcemia, primary hyperparathyroidism

## Abstract

Parathyroid carcinoma (PC) is a rare endocrine malignancy and an uncommon cause of primary hyperparathyroidism. Metastatic disease confers a guarded prognosis with limited systemic treatment options available. We describe a case of a 64-year-old woman with primary hyperparathyroidism secondary to PC. Despite initial surgical resection, the patient relapsed within 6 months with widespread cerebral and skeletal metastatic disease. She developed worsening parathyroid hormone-mediated hypercalcemia that was refractory to escalating doses of cinacalcet and antiresorptive therapy. Molecular genomics identified high tumor mutation burden within the malignant tissue and single-agent nivolumab immunotherapy was administered. After one dose, there was resolution of her refractory hypercalcemia and primary hyperparathyroidism. The patient has tolerated ongoing treatment with 3 weekly cycles of nivolumab. She remains in biochemical remission as of June 2024, which is now 12 months after commencement of nivolumab.

## Introduction

Parathyroid carcinoma (PC) is a rare endocrine malignancy with an incidence of 6 cases per 10 million population (1). It accounts for 0.5% to 2% of cases of primary hyperparathyroidism (2). Metastatic PC carries a guarded prognosis with a median survival of 36 months with refractory hypercalcemia being the leading cause of mortality. We describe a case of refractory hypercalcemia secondary to progressive metastatic PC with a dramatic biochemical and clinical response to nivolumab therapy.

## Case Presentation

**Figure 1.**
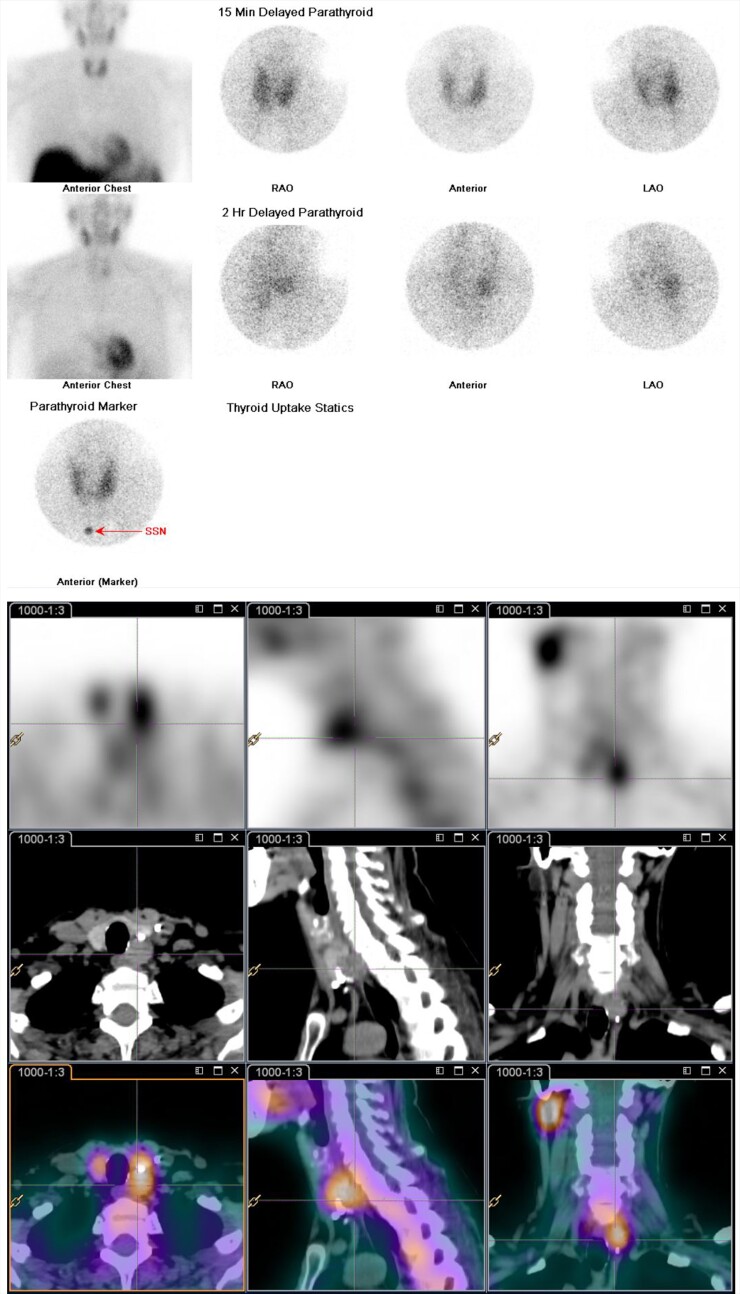
Parathyroid scintigraphy was performed including planar, pinhole, and SPECT/CT imaging following intravenous injection of radiolabeled MIBI in 2021 at first presentation of dysphonia and hyperparathyroidism. There was focal increased MIBI uptake in a 17-mm solid rounded nodule lying posterior to the lower pole of the left lobe of the thyroid. There were a number of focal high attenuating areas lying anterior and inferior to the nodule in keeping with surgical clips. In the 2-hour delayed imaging, there was washout of activity from the thyroid and preferential retention of tracer at the side of the nodule in the left neck. This was in keeping with a left inferior parathyroid nodule. There were no other sites of uptake seen.

A 64-year-old retiree presented with dysphonia in late 2021 and was referred to an otolaryngologist for assessment. She was diagnosed with a left vocal cord palsy due to a nodular lesion arising from posterior to the left lobe of her thyroid gland, appreciable on a computer tomography (CT) scan of her neck. The nodular lesion measured 20 × 16 × 19 mm and abutted the trachea-esophageal groove. It appeared to be lower density than the adjacent thyroid gland, with no appreciable invasion of the esophagus or trachea. Notably, she had primary hyperparathyroidism (parathyroid hormone [PTH] 16.8 pmol/L [158.5 pg/mL], reference 1.6-6.9 pmol/L; [15.1-87.7 pg/mL]) and mild hypercalcemia (albumin-corrected calcium 2.76 mmol/L [11.06 mg/dL], reference 2.10-2.60 mmol/L; [8.4-9.5 mg/dL]). Parathyroid scintigraphy (including planar, pinhole, and single-photon emission computed tomography [SPECT]/CT imaging) identified a 17 mm left inferior nodule with tracer uptake and delayed washout consistent with a parathyroid lesion with no evidence of metastatic disease (Fig. 1.).

Her medical history interestingly included a parathyroidectomy (16 years before presentation in 2005) for a histologically benign left inferior parathyroid adenoma. The adenoma measured 10 × 5 × 5 mm and weighed 285 mg. The patient also had a history of breast cancer diagnosed 9 years before presentation in 2012, which was treated with lumpectomy followed by 3 years of adjuvant tamoxifen treatment. She was also diagnosed with osteoporosis 6 months before current presentation in 2021, (right total hip T-score of −2.0 SD and lumbar spine [L2-L4] T-score of −2.7 SD) and received a single dose of zoledronic acid 5 mg intravenously. She was functionally independent with an Eastern Cooperative Oncology Group (ECOG) score of 0.

## Diagnostic Assessment

**Figure 2.**
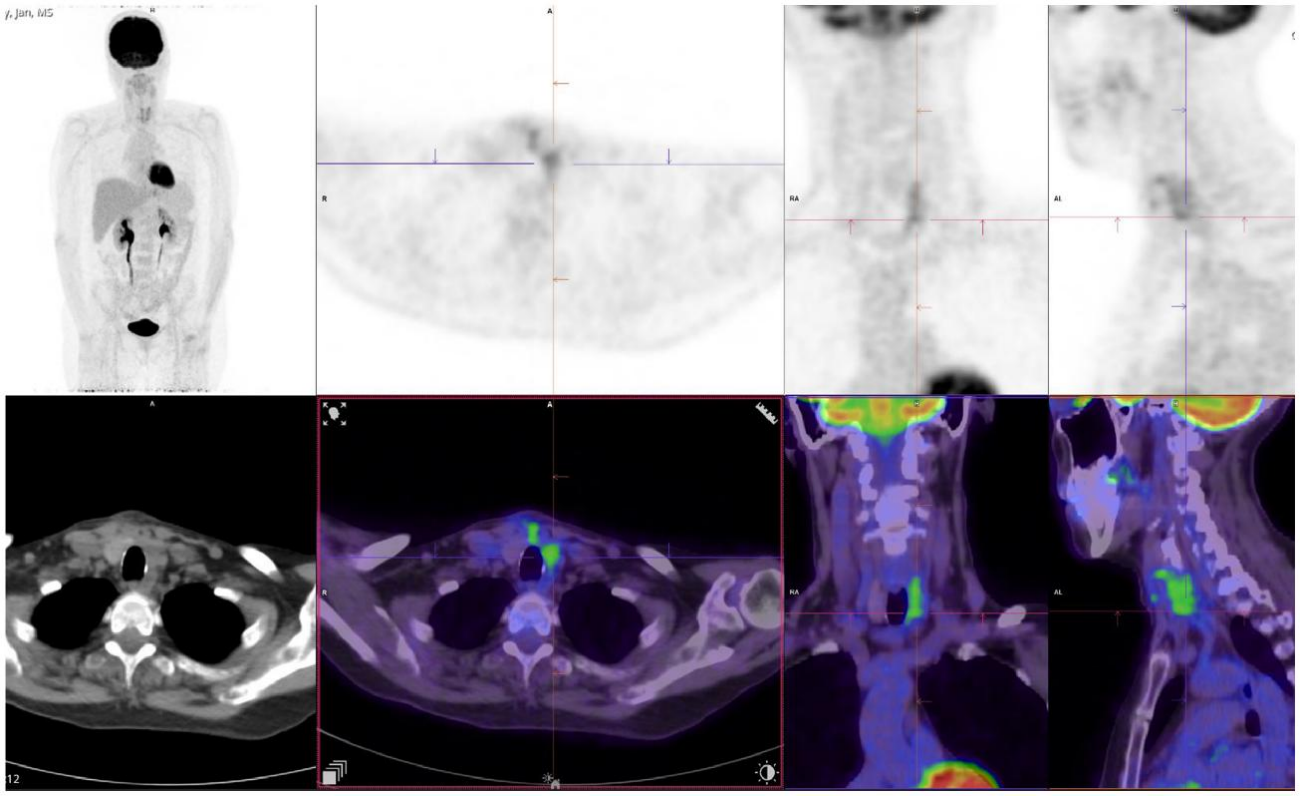
FDG PET-CT performed 1 month following surgical resection of left inferior parathyroid adenoma (February 2022). There was moderate metabolic activity in the left thyroid bed, isthmus, and cervical pretracheal region, and subcentimeter left supraclavicular fossa lymph node, which may be reactive to recent surgery; however, residual disease and nodal metastatic disease could not be excluded. There was no evidence of metabolically active distant metastatic disease. The patient was referred for radiotherapy to the surgical bed following review of images in a multidisciplinary meeting.

A fine needle aspirate of the nodule was performed by the treating surgeon prior to referral to our team. The findings were consistent with a parathyroid neoplasm with staining positive for PTH, MNF116, and GATA3 and negative for TTF1, CD3, CD5, and CD117. She underwent left hemithyroidectomy and left central neck dissection. Intraoperatively, a firm to hard mass measuring 3 cm in length was identified left to the esophageal groove. The mass invaded into the left thyroid lobe and encased the recurrent laryngeal nerve. It was also adherent to but not invading into the esophagus. There was no evidence of residual macroscopic disease. Histopathology revealed a PTH-positive, high-grade carcinoma (Ki67 80%) with evidence of capsular, perineural, and lymphovascular invasion. One of 7 lymph nodes was positive, containing a 2.3 mm deposit with lymphovascular invasion into the adjacent stroma. Her calcium and PTH normalized following surgery. A postoperative fluorodeoxyglucose-18 positron emission and computed tomography (FDG PET-CT) scan demonstrated moderate metabolic activity in the left thyroid bed and a left supraclavicular lymph node (Fig. 2). The increased uptake could have been explained by reactive changes after surgery; however, residual disease could not be excluded. As a result, external beam adjuvant radiotherapy was administered to the thyroid bed (total of 60 Gy over 30 fractions) after discussion in a multidisciplinary team meeting. No somatic mutations (including CDC73, MEN1, RET, or CDKN1B) were identified on genetic testing.

**Figure 3.**
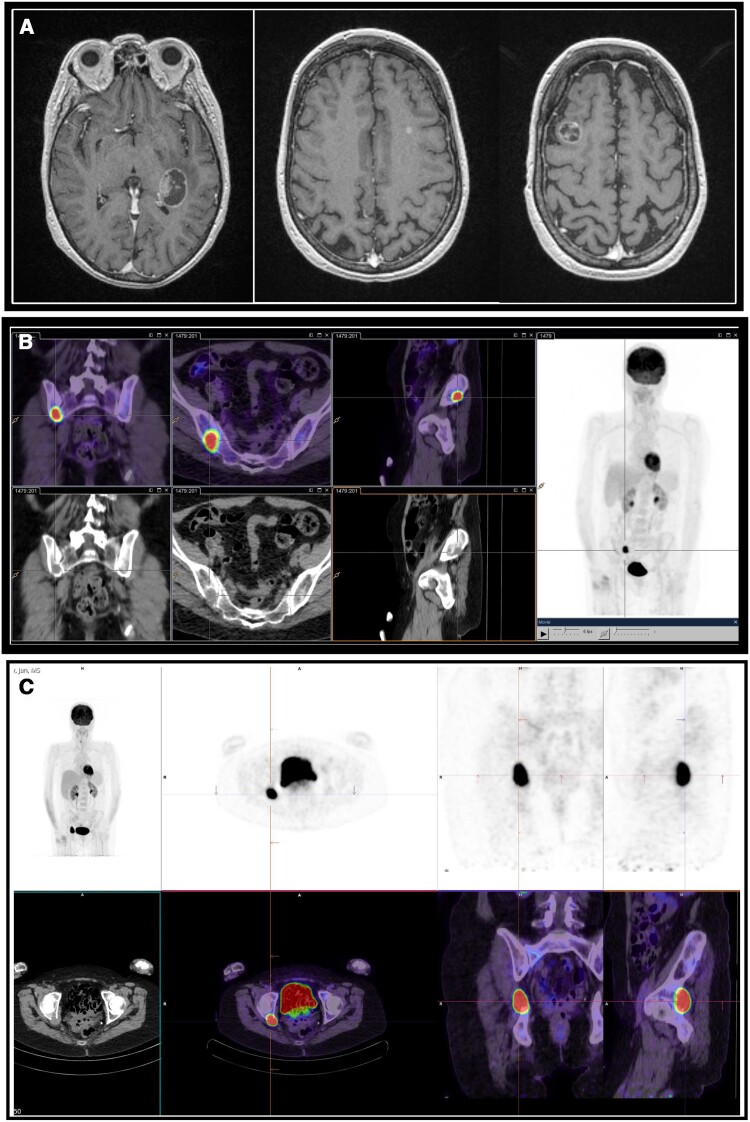
A, B, and C demonstrate sites of metastatic disease. A, MRI head (September 2022) demonstrates a mixed solid and cystic lesion in the left retrolentiform region measuring 30 × 23 mm with moderate surrounding edema and mild mass effect. There is a subcentimeter lesion in the deep white matter of the left frontal lobe (5 mm). There is also a lesion in the right middle frontal gyrus measuring 16 × 14 mm. There were multiple other subcentimeter lesions in the sulcus of the right frontal lobe (4 mm), subcortical right parietal lobe (4 mm), right anterior frontal lobe (3 mm), and left cerebellar hemisphere (7 mm), which are not shown. B, FDG PET-CT (September 2022) demonstrating FDG avid lesion in right ilium. C, FDG PET-CT (February 2023) demonstrating FDG avid lesion in right hip. Radiotherapy was administered 5 fractions (total 30 Gy) to the right ilium, 1 fraction (18 Gy) to 7 brain metastases, 3 fractions (24 Gy) to a large posterior cerebral metastasis, 5 fractions (30 Gy) to the right hip, and 5 fractions (30 Gy) to left ilium (not shown).

Six months later, blood tests revealed disease recurrence with PTH-dependent hypercalcemia (albumin-corrected calcium 2.74 mmol/L [10.98 mg/dL], PTH 14.5 pmol/L [136.8 pg/mL]). Subsequent magnetic resonance imaging (MRI) identified multiple new cerebral metastases and a FDG PET-CT demonstrated an avid lesion in her right ilium (Fig. 3.). Molecular genomic studies revealed a high tumor mutation burden (22.2 mutations/megabase), although low microsatellite instability, suggesting a possible role for immunotherapy. There was also loss of function mutation in MSH2; however, subsequent germline testing was negative for Lynch syndrome.

## Treatment

Shortly after the diagnosis of metastatic disease, radiotherapy was administered, including: 5 fractions (total 30 Gy) to the right ilium, 1 fraction (18 Gy) to 7 brain metastases, 3 fractions (24 Gy) to a large posterior cerebral metastasis, 5 fractions (30 Gy) to the right hip, and 5 fractions (30 Gy) to left ilium. Cinacalcet was commenced. Discussions within a multidisciplinary setting did not identify any systemic treatment options. Given the presence of high tumor mutation burden in the cancer, the patient was referred to multiple clinical trials involving immunotherapy to consider eligibility.

**Figure 4.**
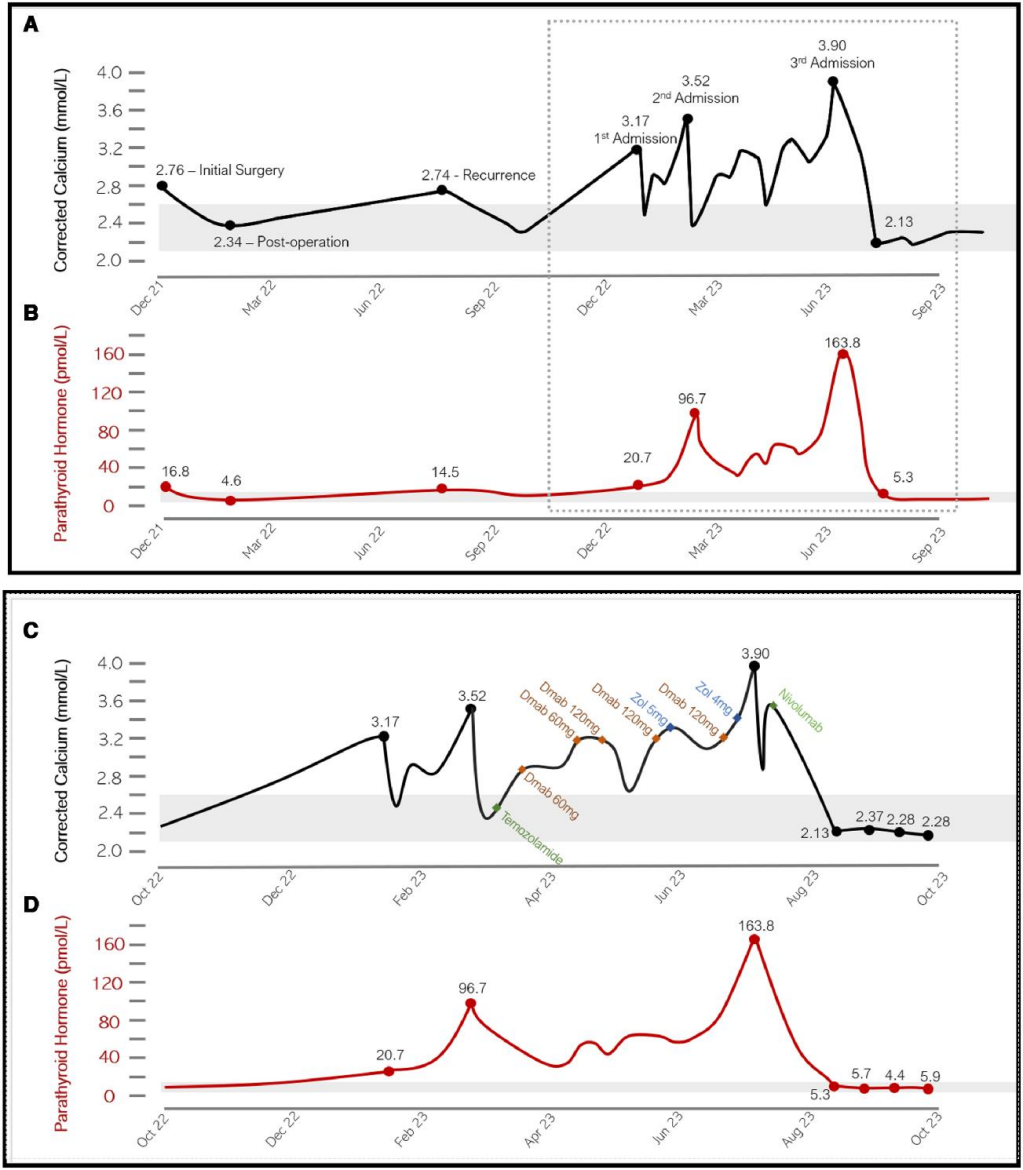
A, This graph demonstrates trends in serum albumin-corrected calcium (A) and parathyroid hormone concentrations (B) and their relation to key events since diagnosis of PC in December 2021. B, This graph narrows in on treatment events occurring within the dotted lines in Fig. 4A. Serum albumin-corrected calcium (C) and parathyroid hormone levels (D) are shown over 12 months from October 2022, shortly after the diagnosis of metastatic PC, to October 2023, following 4 cycles of nivolumab. Note normalization of albumin-corrected calcium and PTH concentrations shortly after nivolumab was commenced. Reference range depicted in gray shading. Abbreviations: Dmab, denosumab; zol, zoledronic acid.

Over the 5 months since her diagnosis of metastatic disease, the patient developed worsening hypercalcemia, refractory to escalating doses of zoledronic acid and denosumab (Fig. 4). Antiresorptive treatment was initially given intermittently for hypercalcemia before intensifying to fortnightly alternating zoledronic acid 4 mg intravenously and denosumab 120 mg subcutaneously. She required 3 hospital admissions for management of hypercalcemia with a peak corrected calcium of 3.82 mmol/L (15.3 mg/dL) and PTH levels consistently above 100 pmol/L (943 pg/mL). Cinacalcet was ceased due to persistent nausea and anorexia. The patient briefly trialed temozolomide, based on a favorable case report (10). Despite this, significant disease progression ensued with new nodal, pulmonary, and skeletal metastases, and the patient was no longer eligible to commence immunotherapy through clinical trials. The patient elected to self-fund nivolumab, an anti-PD1 monoclonal antibody that inhibits immune checkpoint and upregulates T-cell recognition of malignant cells. Nivolumab was chosen as it was the most financially viable option at the time in Australia.

## Outcome and Follow-Up

**Figure 5.**
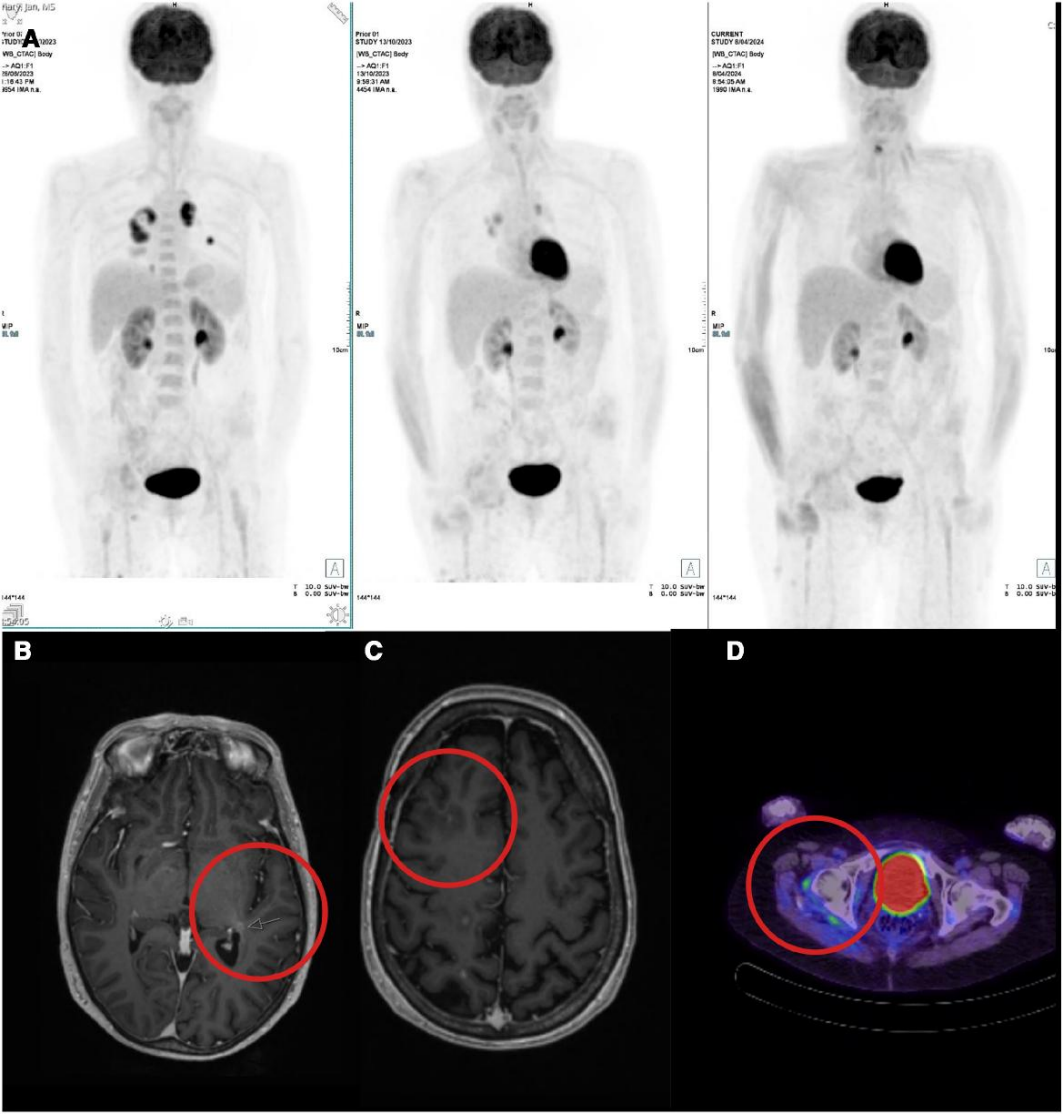
A, B, and C demonstrate areas of disease response with various imaging modalities in April 2024 after 10 months of treatment with nivolumab. A, highlights serial FDG-PET-CT scans through the course of treatment with nivolumab. The first panel on the left was conducted in June 2023 prior to commencing nivolumab. It demonstrated new large nodal and pulmonary metastases in the thorax. The right acetabular metastasis already had marked reduction in avidity, possibly secondary to radiotherapy. The middle panel demonstrates significant but incomplete disease response in the thoracic nodal and pulmonary metastases after 4 cycles of nivolumab (October 2023). The last panel on the right shows an FDG PET-CT scan performed in April 2024, which indicated further metabolic response with very mild metabolic activity in the aortopulmonary region, which may have been due to posttreatment inflammatory change (in view of the area of dense calcification). There was no evidence of metabolically active disease elsewhere. B, MRI scan demonstrates the significant reduction in the lesion in the left retrolentiform region which measured 30 × 23 mm in Fig. 3A. C, MRI scan demonstrates the disease response in the lesion located in right middle frontal gyrus which measured 16 × 14 mm in Fig. 3A. D, demonstrates disease response in the right acetabulum compared to Fig. 3C.

After 1 cycle of nivolumab, the patient's PTH declined from 163.8 pmol/L to 5.3 pmol/L (1545 to 50 pg/mL) and corrected calcium from 3.90 mmol/L to 2.13 mmol/L (15.6 mg/dL to 8.5 mg/dL). Her nausea, lethargy, and constipation resolved. She was prescribed calcium carbonate 600 mg daily and monitored closely for hypocalcemia given her recent treatment with high-dose denosumab and zoledronic acid. FDG PET-CT imaging repeated 4 and 10 months later (Fig. 5) showed significant disease response with near complete resolution of pulmonary nodules and intrathoracic lymphadenopathy. There was also significant disease response in her cerebral metastases (Fig. 5). At the time of article submission (June 2024), the patient has remained in clinical and biochemical remission after commencing regular 3-weekly cycles of nivolumab almost 12 months ago. She has not yet developed any immune-related adverse effects. The plan will be to continue ongoing treatment with nivolumab, given its effectiveness and tolerability.

## Discussion

Challenges exist for both diagnosis and management of PC. PC can be difficult to distinguish from benign parathyroid lesions, both of which cause primary hyperparathyroidism, and definitive diagnosis requires histological analysis. Clinical features thought to favor PC include alkaline phosphatase (ALP) > 285 IU/L, ionized calcium > 1.77 mmol/L (> 7.09 mg/dL), parathyroid lesions > 3 cm or PTH more than 3 times the upper limit of normal (3). Fine needle aspiration is generally not recommended due to the risk of malignant parathyromatosis. Metastatic PC may occur sporadically (90%) or in association with inherited cancer syndromes (10%), including hyperparathyroidism-jaw tumor syndrome, multiple endocrine neoplasia type 1 (MEN1), or multiple endocrine neoplasia type 2A (MEN2A) (3). The loss of tumor suppressor gene called cell division cycle 73 (CDC73) accounts for 70% of sporadic cases. This mutation is rarely found in benign parathyroid adenomas, suggesting different molecular signatures between benign and malignant lesions and PC may arise de novo (4). Theoretically, if there was an adenoma to carcinoma sequence as seen in other types of malignancies, the incidence of PC should be increased in certain populations with high rates of parathyroid hyperplasia, such as in patients with renal failure. There is overlap in the cytomorphological features of PC and benign parathyroid lesions, potentially leading to misdiagnosis (5). Additionally, there are case reports of PC recurring 18 years after initial surgical resection (6, 7). Unfortunately, our patient's original parathyroidectomy specimen from 2005 was discarded, precluding re-analysis. Whether the ultimate diagnosis is one of persistent or primary disease with subsequent metastasis remains uncertain.

PC carries a poor prognosis and there are limited treatment options beyond primary resection. While antiresorptives and calcimimetics can provide initial short-term control of hypercalcemia, disease progression often leads to severe refractory hypercalcemia. Surgery remains the mainstay of treatment of localized disease; however, the optimal surgical approach (localized excision vs en bloc resection) remains unclear (8).

There is limited evidence for radiotherapy, systemic chemotherapy, or immunotherapy, with PC generally considered to be “radioresistant” and “chemoresistant.” There have been 4 cases of reported progression-free survival using 10 months of combined chemotherapy with fluorouracil, cyclophosphamide, and dacarbazine (8). Furthermore, there has been one favorable case report demonstrating the effective use of temozolamide, an oral chemotherapy alkylating agent, in conjunction with surgery, zoledronic acid, and cinacalcet. The patient had relapsed metastatic disease 5 years after her initial surgical resection; however, she was able to achieve remission for at least 17 years after 10 cycles of temozolamide and surgical resection of nodal oligometastatic disease. The parathyroid tumor had high methylguanine-DNA methyltransferase (MGMT) promoter methylation status, which is a known predictor of response to temozolamide (6). Patients who received other chemotherapy regimens experience partial or no response (8).

Evidence for the use of immunotherapy is sparse. There have been 3 case reports from 2004 that investigated PTH immunization, a novel form of immunotherapy to induce antibody formation against human PTH. One Japanese patient died due to disease progression while receiving treatment (9). The other 2 case reports described 24 months and 12 years progression-free survival, respectively (10, 11). These treatments have been largely superseded by modern immunotherapeutic agents.

High tumor mutational burden (> 10 mutations per megabase) can identify patients who are more likely to respond to immunotherapy. In a large multicenter trial of 790 patients with less-common solid organ tumors, 29% of patients with high tumor mutation burden had an objective response to pembrolizumab, compared to 6% of patients without high tumor mutation burden (12).

There have been 2 case reports of checkpoint immunotherapy for the treatment of metastatic PC. The first case report (13) described a patient with relapsed metastatic PC 12 years after the primary diagnosis and local resection. She had high tumor mutation burden, similar to our patient and received pembrolizumab (anti-PD-1 antibody). There were subsequent surgical resections of oligometastatic disease in abdominal lymph nodes. The patient had biochemical remission up to 3 years later at the time the report was published. The second case report detailed a patient with Lynch syndrome, defined by microsatellite instability which generates tumor mutation burden. Pembrolizumab administered over 4 months (5 cycles), achieved a partial radiological response (60% reduction in pulmonary metastases). There was a complete biochemical response (normalization of serum calcium and PTH) before it was discontinued due to severe immune-related colitis. Despite cessation, disease volume and biochemistry remained stable 24 months later (3, 14).

To our knowledge, this is the first case report to detail the use of nivolumab for the treatment of metastatic PC. Immunotherapy may be an emerging treatment option, informed by individual cancer genomics.

## Learning Points

- PC is a rare endocrine malignancy that causes PTH-mediated hypercalcemia.
- Consider PC as a diagnosis in cases of primary hyperparathyroidism with ALP > 285 IU/L, ionized calcium > 1.77 nmol/L (> 7.09 mg/dL), parathyroid lesions > 3 cm, or PTH > 3x ULN.
- Limited treatment options exist for PC, particularly for metastatic disease, which carries a guarded prognosis.
- Management of hypercalcemia due to PC is with antiresorptives and calcimimetics; however, break-through hypercalcemia will invariably occur with disease progression.
- Immunotherapy may be a treatment option for metastatic PC depending on cancer genomics.

## Data Availability

The data that support the findings of this report are available on request from the corresponding author (B.T.). The data are not publicly available as it contains information that could compromise patient privacy.

## Abbreviations

CT: computed tomography
FDG: fluorodeoxyglucose
MRI: magnetic resonance imaging
PC: parathyroid carcinoma
PET: positron emission tomography
PTH: parathyroid hormone
SPECT: single-photon emission computed tomography
